# Supplementary material for: Long-Term Changes to the Microbiome, Blood Lipid Profiles and IL-6 in Female and Male Swedish Patients in Response to Bariatric Roux-en-Y Gastric Bypass
Source: Nutrients. 2024 Feb 9;16(4):498. doi: 10.3390/nu16040498 (PMC10891850; doi:10.3390/nu16040498)
Supplement: Supplementary file 1 [file nutrients-16-00498-s001.zip › Figure S1. Visual abundance of gut microbiome._Nutrients.pdf]

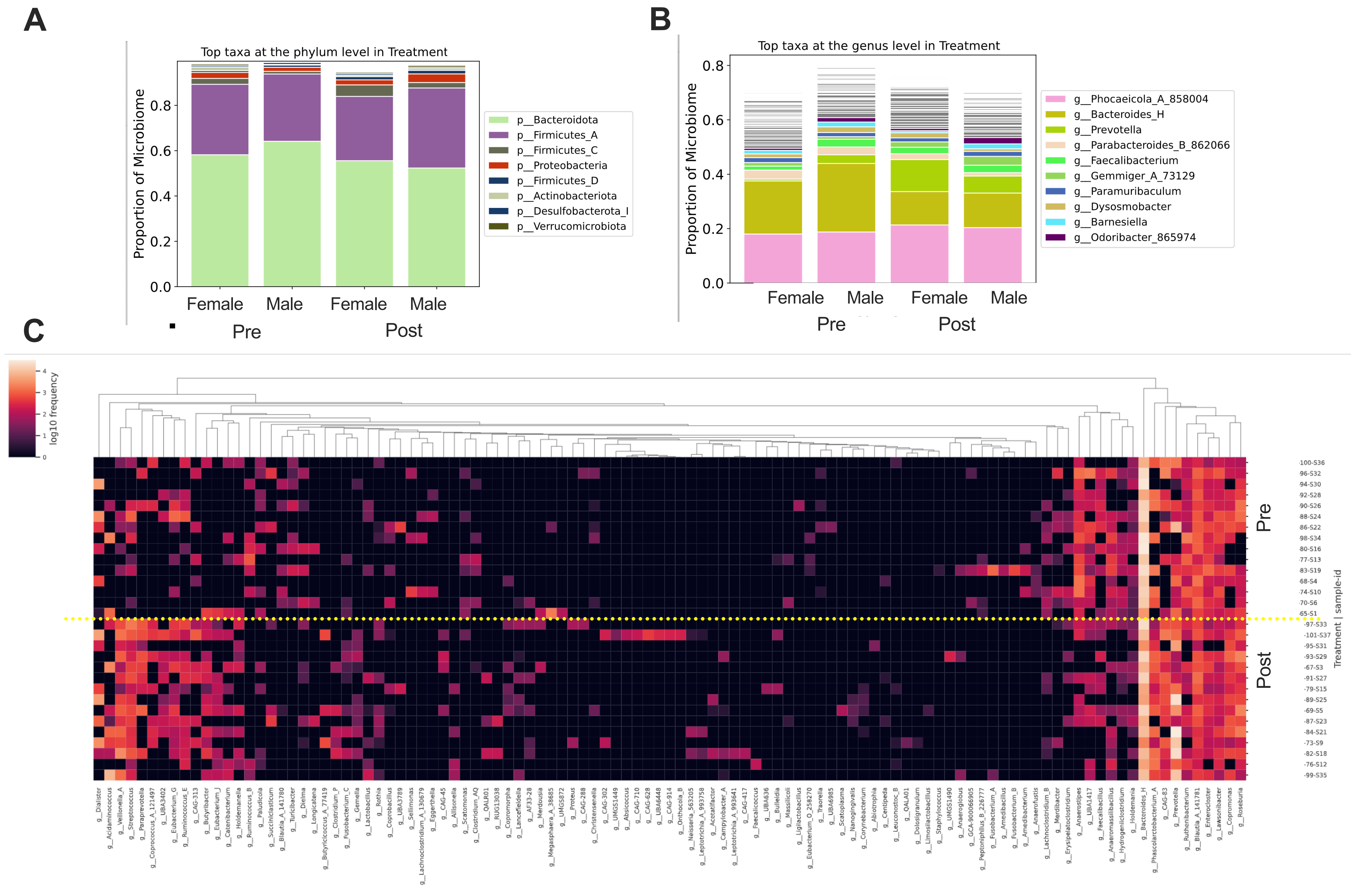

Figure S1. The visual abundance of all bacteria at phylum (A) and genus levels (B) per group and in individual patients (C) before (pre-) and after (post) surgery.
